# Hierarchical Hybrid Electrodes (HHE) for Enhancing the Performance of Water Electrolysis Systems

**DOI:** 10.3390/nano16090500

**Published:** 2026-04-22

**Authors:** Sanskar Shrestha, Sathvik Peddamalla, Wenhu Wang, Sharmila M. Mukhopadhyay

**Affiliations:** 1 Department of Mechanical Engineering, Maine College of Engineering and Computing, the University of Maine, Orono, ME 04469, USA; sanskar.shrestha@maine.edu; 2 Frontier Institute for Research in Sensor Technologies, the University of Maine, Orono, ME 04469, USA; sathvik.peddamalla@maine.edu

**Keywords:** hierarchical nano electrode structures, carbon nanotubes, nanocatalysts, oxygen evolution reaction, hydrogen evolution reaction

## Abstract

Electrolysis of water is a promising emission-free approach of hydrogen production, making water electrolyzers important for many renewable energy systems. Electrochemical electrodes enriched with nanocatalysts can significantly advance such technologies, but the use of nanomaterials, deployed as packed powders or painted films, is generally limited by durability and reusability challenges. To overcome these deficiencies, we have fabricated hierarchical hybrid electrode (HHE) monoliths comprising carpet-like arrays of multiwalled carbon nanotubes covalently bonded to porous reticulated carbon foams that are further functionalized with strongly attached nanocatalysts. This paper presents our investigation of HHE materials with CNT carpets and palladium nanoparticle (PdNP) catalysts in two key electrolysis reactions: hydrogen evolution reaction (HER) and oxygen evolution reaction (OER). Their performances in different electrolytes have been evaluated using cyclic voltammetry, linear sweep voltammetry and Tafel analysis. This architecture provided multi-faceted advantages, and the contribution of each nanocomponent in the monolith has been analyzed. The presence of Pd-NP in the HHE also improved the electrode’s tolerance to Cl^−^ ions, which is very promising for saline water electrolysis. These studies indicate that the HHE architecture of electrochemical electrodes can be a versatile and tunable option for future electrochemical systems relevant to renewable energy applications.

## 1. Introduction

Generation of hydrogen from water is an important technology that can provide sustainable energy [1,2,3,4] at the present time when concerns about fossil fuels and global warming are rising globally [5,6]. Advanced customized water electrolysis systems can use abundantly available water to create a clean energy fuel like hydrogen and is especially attractive because it can be powered on-site by renewable energy sources [7,8,9]. Water electrolysis splits water into hydrogen and oxygen via two half reactions: the oxygen evolution reaction (OER) at the anode and the hydrogen evolution reaction (HER) at the cathode. Several studies have suggested a possible reaction mechanism [10,11]; overall, there are two half reactions to consider in alkaline medium (KOH), in which the OER and HER half reactions are

4OH^−^ → O_2_ + 2H_2_O + 4e^−^ (1)

2H_2_O + 2e^−^ → 2OH^−^ + H_2_ (2)

And in acidic medium the OER and HER half reaction are

2H_2_O → O_2_ + 4H^+^ + 4e^−^ (3)

2H^+^ + 2e^−^ → H_2_ (4)

In general, in neutral electrolyte, OER half reaction is expected to follow the pathway of acidic condition, and HER half reaction is expected to follow that of alkaline medium. An important strategy to enhance the performance and cost-effectiveness of water electrolyzers is to develop more advanced electrodes for easier and faster electrochemical reactions under practical service conditions. Important aspects of electrode design include (i) maximizing the chemical and structural stability, (ii) increased specific surface area of the electrode for increased interaction with the electrolyte, (iii) improved fluid transport at the interface, and (iv) incorporation of surface-active components such as electrocatalysts to promote and control electrochemical reactions [12].

Graphitic carbon is an attractive electrode material due to its chemical inertness and low density combined with high electrical conductivity. We have used a porous form of a commercially available carbon structure, reticulated vitreous carbon, or RVC, as the base substrate for our water electrolyzer electrodes. RVC combines the inherent advantages of carbon solids with an interconnected porous geometry that provides abundant fluid transport pathways.

Nanostructures of graphitic (sp^2^) carbon, especially carbon nanotubes (CNTs), have gained attention as promising materials for several electrochemical applications [13,14,15,16,17]. CNTs are known for unusually high electrical conductivity along the axial direction with high mechanical strength and chemical stability. In addition, they have ultra-high surface-to-volume ratio which provides a high concentration of surface interaction sites for electron transfer, and the ability to support functional/catalytic nanoparticles as needed [18,19]. Moreover, these structures can be further customized as needed to support nanoparticles for catalysts or other electrochemical functions. Acting as excellent anchors, CNTs facilitate uniform dispersion of catalysts, enhance charge transport, and provide a synergistic effect that optimizes the electrode architecture for efficient oxygen evolution.

However, loose CNTs are not directly usable in practical reusable electrodes. When encapsulated in another phase, the surface activity is compromised. When used as compacted physical aggregates, the likely disintegration with use in water poses risk of secondary contamination with nanomaterials. These challenges have limited the widespread use of nanomaterials in practical electrochemical applications. Integrating CNTs into durable electrode structures without compromising their surface availability would be ideal but has been challenging so far [20,21,22]. Our research team has addressed this challenge by designing and fabricating multiscale carbon electrodes where carpet-like arrays of multiwalled carbon nanotubes (MWCNTs) have been covalently bonded to the surface of RVC foam. The resulting hierarchical hybrid nano electrode combines the advantages of CNT surface with the porous and conductive framework of RVC foam that together provide a very high density of active sites for electrochemical exchange and significantly enhanced fluid flow to boost overall performance.

In water electrolysis, several metals and metal oxides have been known to serve as effective electrocatalysts [23,24,25]. This includes noble metals such as platinum, palladium, iridium and rhodium. Among those metals, palladium stands out as uniquely promising due to its low activation energy and strong affiliation with hydrogen [15,16,17,26,27,28,29,30]. In this study, we have therefore attached palladium nanoparticles (Pd-NP) to the CNT surfaces bonded to RVC and investigated their performance.

The combined material constitutes the hierarchical hybrid electrode (HHE). This design principle provides a platform for precise integration of several materials into one working electrode. This study analyses the electrochemical properties of the HHEs in HER and OER. We have studied how the CNTs and palladium nanoparticles impact the performance of the electrodes using electrochemical techniques like cyclic voltammetry (CV), linear sweep voltammetry (LSV), and Tafel analysis (TA), where the surface-active sites were studied via studying the electrical double layer capacitance (EDLC) using CV [31], the exchange current and potential for HHEs were studied via Tafel plot analysis (TPA), and the HER and OER half reactions were characterized using LSV and TA. The findings from this study could provide valuable insights for designing more efficient and durable electrodes, contributing to the development of better energy conversion and storage technologies.

## 2. Experimental

### 2.1. Chemicals and Equipment

The chemical reagents used in this study were of analytical grade. These include xylene (Millipore Sigma, Burlington, MA, USA), ferrocene (99% Alfa-Aesar Ltd., Ward Hill, MA, USA), KOH (85% ACS reagent, Sigma Aldrich, St. Louis, MO, USA), H_2_SO_4_ (18M, Fisher Scientific, Hampton, NH, USA), KCL (Fisher Chemical, Hampton, NH, USA), HCl (37% Acros Organics Ltd., Waltham, MA, USA), tetraamminepalladium(II) nitrate (TAPN) (99%, 5% Pd, Alfa Aesar, Ward Hill, MA, USA), and methanol (99%, Fisher Chemical, Hampton, NH, USA). Other materials used were Deionized (DI) water (FIRST, Orono, ME, USA) and laboratory grade gases that include hydrogen, argon and oxygen. The reticulated vitreous carbon foam (RVC) used was 80 ppi (pores per inch) grade obtained from Ultramet Inc., Pacoima, CA, USA. Surface chemistry of the samples was analyzed by X-ray photoelectron spectroscopy (XPS) using a SPECS PHOIBOS HSA 3000 Plus hemispherical energy analyzer (Berlin, Germany). The magnesium (Mg) was used as the anode to generate Mg K alpha (1253.6 eV) X-rays for analysis. Microstructural analysis was conducted using Thermal Fisher Scientific Helios 5 CX scanning electron microscope (Hanover, NH, USA). A HI2002 edge pH meter from (Hanna Instruments, Woonsocket, RI, USA) were used for pH measurements and the electrochemical measurements were carried out using a CHI-650D potentiostat (CH instruments, Austin, TX, USA).

### 2.2. Synthesis of the Hierarchical Hybrid Rvc-Cnt Structure

**Figure 1:**
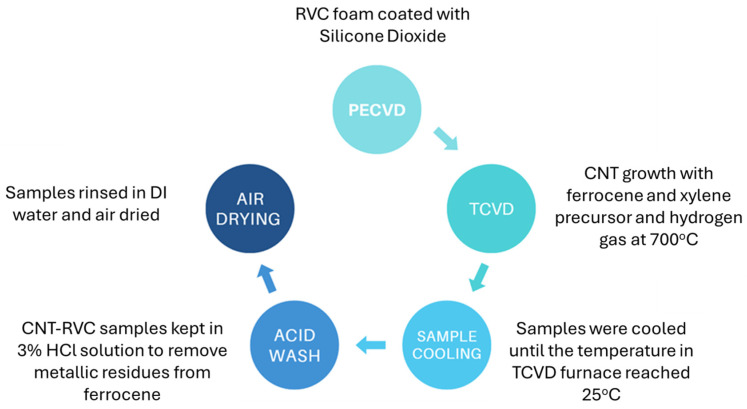
Schematic representation of CNT-RVC synthesis.

Reticulated vitreous carbon (RVC) was used as a base for the hybrid structure. RVC foam used in this study has a porosity of 80 pores per inch (ppi) and an average pore size of 317.5 μm. This grade of RVC has a specific surface area of 0.1 m^2^/g and exhibits 97% porosity. This pore size is deemed to enable adequate gas passage and the synthesis of CNTs, through chemical vapor deposition (CVD) on the pore walls [32]. Synthesis of CNTs on RVC was executed using a multi zone CVD furnace reactor which was purchased from (MTI Corporation Ltd., Richmond, CA, USA) as seen in Figure 1. A mixture of ferrocene and xylene was used as the catalyst and the carbon source. This mixture was injected into the pre heated zone at 380 °C. The evaporated mixture then traveled to a reaction zone kept at 700 °C, kept in an Ar/H_2_ environment. To investigate the impact of CNT carpet length on electrochemical properties, two growth times have been selected: 1 h (CNT1-RVC) and 3 h (CNT3-RVC). Previous studies in this group have systematically investigated CNT growth rates and other parameters on model flat surfaces using the exact same method used here, as shown in the reference [32,33]. These had confirmed that the CNT growth rate was about 1.67 μm/min. This indicates that in our samples, the average CNT lengths are expected to be about 100 μm for the 1 h CNT and about 300 μm for the 3 h CNT. After the CNT growth was completed, the furnace was allowed to cool down at room temperature in a reduced flow of Ar. The structural morphology of the RVC-CNT structure was studied using scanning electron microscopy (SEM). The CNT growth times have a direct influence on the length of the CNT carpets. All electrodes used for electrochemical measurements were cut into cross sections of 3.0 mm diameter with a height of 7.0 mm, the geometric lateral surface area of these electrodes is 0.7 cm^2^.

### 2.3. Incorporation of Palladium Nanoparticles (PdNPs)

**Figure 2:**
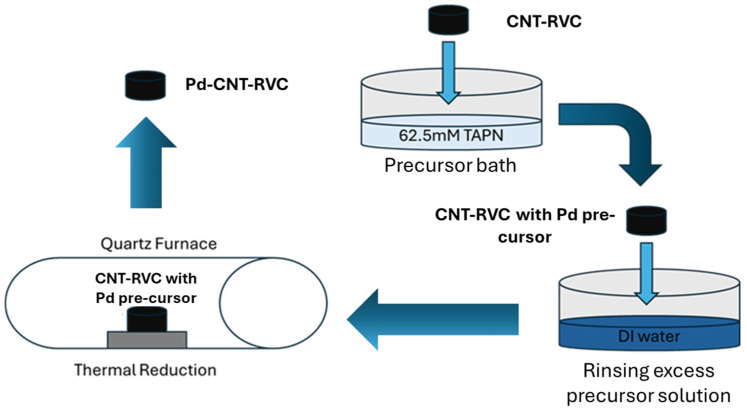
Schematic representation of Pd-CNT1-RVC synthesis.

To investigate the impact of palladium nanocatalysts on electrochemical properties, palladium nanoparticles (PdNPs) were deposited directly on 1 h carbon nanotube-grown RVC foams (CNT1-RVC). This was done through infiltration of inorganic precursor followed by controlled reduction in a controlled environment. The details of this method for synthesizing PdNPs on solid carbon-based substrates have been documented in previous publications [34,35,36]. In brief, a 62.5 mM solution of Tetraamine palladium (II) nitrate (TAPN) was used as the metal precursor. The CNT-RVC hybrid electrodes were immersed in a diluted TAPN solution for a set duration. After immersion, the electrodes were removed from the solution, and any excess solution was washed off by briefly dipping the sample in water. The subsequent thermal reduction was carried out in a programmable quartz furnace, the schematic illustration of Pd nanoparticle deposition is shown in Figure 2. The resulting Pd-CNT1-RVC hybrid material was then used as the working electrode for the studies.

### 2.4. Electrochemical Characterization

The electrochemical (EC) performance of HHEs was evaluated using a three-electrode setup comprising a platinum wire counter electrode and an AgCl reference electrode on a CHI 660E potentiostat. Prior to EC analyses, all samples were cleaned with 3% HCl solution and rinsed repeatedly in methanol and water. Electrolyte solutions were purged with nitrogen gas before testing. Current responses were normalized to the electrode’s geometric surface area (0.7 cm^2^). Cyclic voltammetry (CV) and Tafel plot measurements were conducted to estimate electric double-layer capacitance (EDLC) and exchange current density and equilibrium potential in 0.02 M KCL at a scan rate of 20 mV/s. Linear sweep voltammetry (LSV) with 100% iR compensation at a scan rate of 10 mV/s was performed. For investigating the oxygen evolution reaction (OER), the potential was swept from 0 to 2 V (vs. AgCl), and for studying the hydrogen evolution reaction (HER), the range was 0 to −2 V (vs. AgCl). These tests were carried out in three electrolytes: 0.2 M each of KCl, KOH, and H_2_SO_4_. Electrolyte concentration was chosen as 0.2 M, after initial trials in different concentrations of electrolytes ranging from 0.2 M to 5 M. Those results are shown in the Supplementary Document in Figures S1 and S2. It was noted that at higher electrolyte concentrations, gas bubbles were observed on the electrode during the HER and OER experiments, negatively impacting our data collection and accuracy. Since the goal of this study is to develop an in-depth understanding of the CNT and catalyst nanostructures in the hierarchical hybrid electrode (HHE) material in different electrolytes, we selected the lower concentration (0.2 M) for detailed analysis, which would show more accurately measurable currents for analysis. The pH was maintained at 0.7, 7.0 and 13.4, respectively. Chronopotentiometry (CP) experiment was carried out to test the HHE long-term durability. The current density was held at −10 mA/cm^2^ and 10 mA/cm^2^ while the voltage over time was monitored. The onset potentials were derived from LSV potentials taken to reach 10 mA/cm^2^ and Tafel slopes calculated from the linear regions around these onsets. The LSV plots’ potential ranges were adjusted for each electrolyte to report clear signals and reduce noise due to technical limits. All the resulting potential inputs for HER and OER LSV plots, as well as CV plots for EDLC, were converted from Ag/AgCl to the reversible hydrogen electrode (RHE) scale using Equation (5), where pH is the electrolyte pH and E(AgCl) = 0.197 V vs. RHE at 25 °C:

E(RHE) = E(AgCl) + 0.197 + (0.059 × pH)(5)

## 3. Results and Discussion

### 3.1. Material Characterization

#### 3.1.1. Electrode Morphology

**Figure 3:**
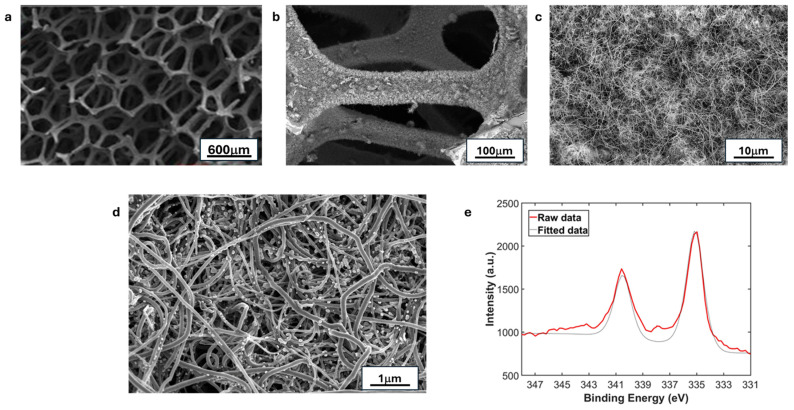
SEM images illustrating the morphology of (**a**) pristine RVC foam, (**b**) RVC surface after CNT growth (low magnification), (**c**) RVC surface after CNT growth (high magnification), and (**d**) palladium nanoparticle attachment on the carbon nanotube carpet. (**e**) XPS spectrum of metallic Pd nanoparticles on the RVC-CNT surface.

Microstructural analyses of the electrodes are shown in Figure 3. Figure 3a shows bare RVC foam and Figure 3b shows RVC foam coated with CNT1-RVC. It can be seen from Figure 3b that the CNTs cover the RVC foam surface completely with a carpet-like layer. Figure 3c shows higher magnification image of the CNT surface, and Figure 3d shows the same surface after PdNP attachment. These prove that the Pd-CNT-RVC hierarchical hybrid electrode (HHE) contains uniformly distributed Pd nanoparticles on uniform carpet-like array of CNT on the walls of the RVC struts. Agglomeration was barely observable throughout the sample, signaling that the Pd nanoparticles are anchored individually on the CNT surface. In previous reports by this group using the same deposition process, it was seen that the number of Pd-NPs on CNT-coated RVC foam was found to be around 7.1 × 10^4^ PdNPs/${\mu m}^{2}$. It was also shown that the Pd-NP were anchored on the CNT walls, with particle diameters ranging from 10 to 25 nm [35]. Figure 3e shows the XPS fine scan of the Pd nanoparticles, with peaks at 335.12 eV and 340.61 eV are characteristic of metallic Pd. The peak separation between Pd*3d*_5/2_ and Pd*3d*_3/2_ is also measured at 5.37 eV and can be attributed to zero-valent metallic Pd.

#### 3.1.2. Electrochemical Characterization

**Figure 4:**
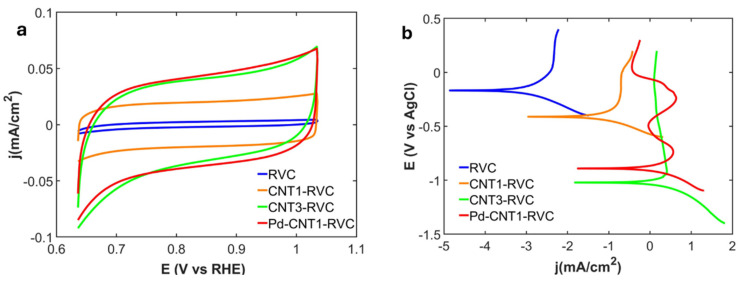
(**a**) CV taken at a scan rate of 20 mV/s, (**b**) Tafel plot taken at scan rate of 10 mV/s in 0.02 M KCL.

- **Cyclic voltammetry (CV):** The CV curve in 0.02 M KCL electrolyte is shown in Figure 4a and highlights the impact of surface features on the electrode’s capacity for charge storage, and their effects on the double-layer capacitance.

***Impact of CNT length:*** The electric double-layer capacitance (EDLC or C_dl_) for the RVC, 1 h grown carbon nanotubes (CNT1-RVC), and 3 h grown carbon nanotubes (CNT3-RVC) are 107.14 mF/cm^2^, 982.14 mF/cm^2^ and 1794.66 mF/cm^2^ respectively. This indicates that attachment of CNT nano-carpets has very significant increase in electric double-layer capacitance (EDLC). This increase can be attributed to the increased specific surface area of highly conducting CNTs providing many active sites, which are critical for the formation of the electrical double layer, leading to an overall capacitance increase compared to pristine RVC. While increasing CNT length increases EDLC, it may not be possible to create a direct linear quantitative relationship between the two, because increasing CNT length is accompanied by entanglement of CNT strands in the carpet, which can block some surface sites and impact fluid diffusion [37,38]. The improvement in EDLC due to varying lengths of CNT determines that charge storage is dependent on the surface area of the electrode itself.

***Impact of Pd:*** Electrodes enriched with CNT and further incorporated with PdNP showed additional increase in EDLC. The EDLC for Pd-CNT1-RVC was seen to be 1946.43 mF/cm^2^, which even surpassed the EDLC of 3 h grown CNT electrode (CNT3-RVC). This improvement indicates that the presence of PdNP can significantly enhance the electrical double layer by providing increased density of surface-active sites.

b. **Tafel analysis:** As mentioned earlier, Tafel plots are highly valuable for evaluating the electrochemical performance of electrodes, as they provide insights into the reaction energy barriers (which determine how easily a reaction can begin) and the reaction rate (which reflects the kinetic efficiency) [39]. The results from Tafel analysis of our HHE materials are shown in Figure 4b, and the exchange current densities and equilibrium potential are tabulated in Table 1 and discussed below.

***Impact of CNT length:*** The hybrid CNT-enhanced RVC structures exhibit lower overpotentials, indicating that the electrodes can operate effectively at lower energy inputs, and provide higher current densities, implying that reaction rates will be faster. This could be attributed to the CNTs providing more active sites to interact with the ions in the electrolyte, while offering ultra-high conducting pathways for electron transport. These findings suggest that long CNT carpets can lead to substantial improvements in onset as well as electrochemical reaction rates of these electrodes.

***Impact of Pd:*** The Pd-CNT1-RVC sample shows significant reduction of overpotential and increase in current density compared to CNT1-RVC. This means lower energy is required for surface interactions in the presence of PdNP and the charge transfer rates are also increased. This implies that even without increasing CNT length, as shown in the last results, attachment of electrocatalytic nanoparticles can also enhance the electrochemical properties in multiple ways.

In summary, these studies show that there are two strategies for increasing the overall electrochemical properties of porous carbon electrodes. One approach is to add CNT carpets, adjusting their length as needed. The other is to enhance the properties of CNTs of a given length by attaching nanoparticles to them.

### 3.2. Study of Water Electrolysis

#### 3.2.1. Oxygen Evolution Reaction (OER)

In alkaline media, OER is dependent on OH^−^ ions and CNTs are excellent materials for OH^−^ adsorption [40,41]. This results in a faster first step in alkaline environment, which is forming *OH. Gibbs free energy of OER in alkaline media can be derived from Equations (6) and (7) and will be dependent on the OH^−^ concentration as shown below.

(6) $$4OH^{-}\to O_{2}+2H_{2}O+4e^{-}\;\;\;\;Q=\frac{\left[pO_{2}\right]}{{\left[OH^{-}\right]}^{4}}$$

(7) $$\Delta G_{O_{2}/H_{2}O}=\Delta G^{0}+RT\ln\left(\frac{pO_{2}}{{\left[OH^{-}\right]}^{4}}\right)$$

This means that in KOH, the Gibbs free energy of the OER should also be theoretically lower, provided there is a higher amount of OH^−^ ions.

On the other hand, Gibbs free energy in acidic and neutral media, OH^−^, needs to be extracted from the water molecules as seen in Equation (8).

(8) $$H_{2}O\;(l)+*\to *\mathrm{OH}+H^{+}+e^{-}\;\;Q1=\frac{\left[*OH\right]\left[\left[H^{+}\right]\right]}{\left[H_{2}O\right]\left[*\right]}$$

**Figure 5:**
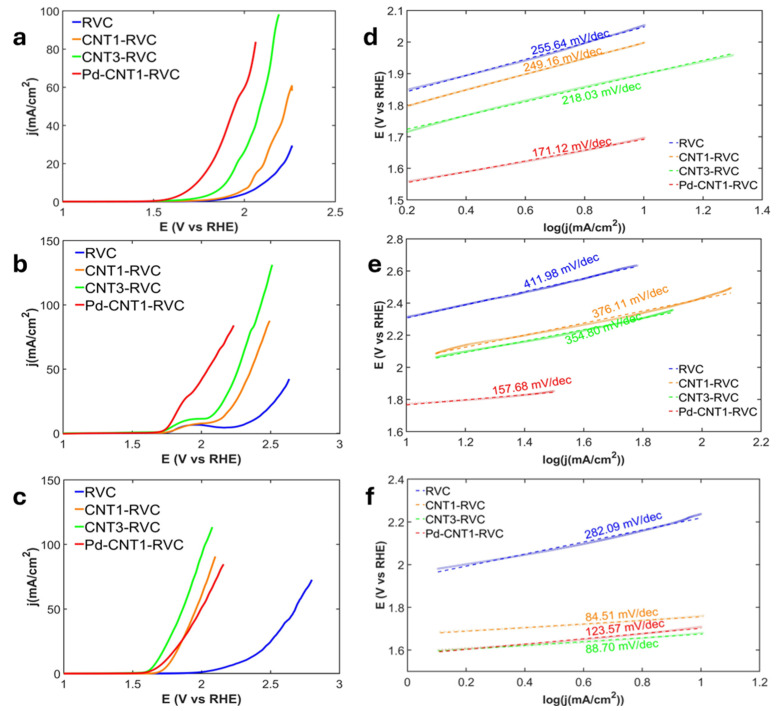
LSV curves of CNT-RVC based electrodes for OER, taken at a scan rate of 10 mV/s in 0.2 M; (**a**) H_2_SO_4_, (**b**) KCL, and (**c**) KOH. Tafel plots in 0.2 M; (**d**) H_2_SO_4_, (**e**) KCl, (**f**) KOH, extrapolated from the LSV curves.

Figure 5a–c present the linear sweep voltammetry (LSV) curves of CNT-based electrodes tested in 0.2 M strength of H_2_SO_4_, KOH, and KCl electrolytes respectively. Table 2 summarizes the comparative performance of CNT-based electrodes towards the oxygen evolution reaction (OER), highlighting key metrics such as onset potential and Tafel slope across different electrolytes. This data is averaged over six repeated experiments.

***The key findings from these results can be summarized as follows:***

While the onset potential and Tafel slope of a given electrode varies from electrolyte to electrolyte, as expected, our focus has been on comparison of onset potentials of different electrodes in each given electrolyte.

***Analyses of onset potential (E_on_)***: It is notable that in all environments, Pd-CNT1-RVC samples clearly reduce the onset potential for OER, indicating that they can reduce the energy barrier to reaction start. Earlier studies have reported that the platinum-group metals have shown beneficial catalytic effect on OER across a broad span of pH [42]. Another notable observation is that in alkaline condition, the CNT1-RVC electrode provides a clear drop in onset potential, due to the abundance of hydroxide in this medium and the CNTs being suitable hosts for the OH^−^ ions.

***Tafel slope analyses to predict variations in reaction rates (β):*** Figure 5d–f present the Tafel slopes of OER on RVC-CNT in 0.2 M solutions of H_2_SO_4_, KCL and KOH respectively. The respective pH values were 0.7, 7.0 and 13.4. The Tafel slopes (β) calculated were in terms of mV/dec.

***Impact of CNT on OER in alkaline medium:*** From Table 2, in KOH electrolyte (which is expected to follow Equation (3)), adding CNTs to the RVC electrode causes a clear drop in the Tafel slope, which indicates a faster reaction. In alkaline environment, the OER depends on the availability of OH^−^ ions, which promotes the first step, and CNT surfaces provide excellent sites for their adsorption [40,41].

***Impact of palladium on Tafel slope:*** In neutral and acidic electrolytes, the presence of PdNP is seen significantly reducing the Tafel slope compared to all other electrodes, indicating that the extra surface-active sites can be useful in speeding up the charge transfer rates. The exception here is the alkaline electrolyte, where the CNT carpets alone (CNT1-RVC and CNT3-RVC) show significantly higher rates as mentioned earlier. In this case, Pd-CNT1-RVC shows a reduced Tafel slope compared to CNT1-RVC, which can be attributed to the fact that some of the CNT surface sites (which are super-adsorbents for OH^−^ ions) would now be masked by Pd.

***Impact of Pd NP on chlorine-ion tolerance:*** Another important conclusion emerges from these studies in KCL medium. Pd nanoparticles may increase the tolerance of water electrolysis systems to chlorine ions and enhance their performance in saline environments. As shown in Figure 5b, (KCl electrolyte) RVC, CNT1-RVC and CNT3-RVC electrodes show a clear secondary peak in the vicinity of 1.9 V vs. RHE due to the chlorine evolution reaction in the presence of chloride. In contrast, the Pd-CNT1-RVC electrode completely bypassed this secondary peak by initiating the OER process at lower energy. This indicates that the HHE with Pd-CNT1-RVC architecture may enable water electrolyzers to bypass the challenging Cl^−^ evolution complications in future water electrolysis systems.

Earlier studies reported on scaled-up water electrolysis systems pointing to the advantages of Pd. For instance, it was reported that in neutral- to high-pH environments, Pd had shown effective corrosion resistance from chlorine during long-term stable seawater electrolysis, because the natural interaction between Pd and Cl^−^ helps prevent corrosion of active sites and repels Cl^−^ through the common-ion effect, which improves OER selectivity and prolongs the electrode’s durability [43]. Our observation is that Pd in HHE can even suppress the Cl^−^ evolution, adding to its promise.

In summary, for HHE materials in alkaline media, the CNT1-RVC (84.51 mV/dec) and CNT3-RVC (88.7 mV/dec) electrodes show the lowest Tafel slope values across all experiment groups, which are 84.51 mV/dec and 88.7 mV/dec, respectively. This is attributed to OH^−^ adsorption on the CNT surface making them more interactive. In non-alkaline media (lower pH), Pd catalyst in Pd-CNT1-RVC showed a lowering of Tafel slope. This is not the case in alkaline medium, where Pd increases the Tafel slope, which may be because it covers up some of the CNT sites, hence reducing OH^−^ adsorption. Additionally, the Pd-CNT1-RVC was seen to clearly bypass the Cl^−^ evolution reaction during OER.

These electrolyte-specific trends in Tafel slope performance point to the importance of tailoring both the electrode’s composition specific to each reaction environment, paving the way for strategic use of nanomaterial–CNT–substrate hybrids to optimize OERs.

***OER Performance comparison with others reported in the literature:*** Some earlier publications [44,45,46,47] have also shown that the incorporation of CNT–nanocatalyst complexes can lead to significant improvements in Tafel slope values for the OER in alkaline medium and have highlighted the crucial role of CNTs not only as a conductive support but also as an active component that contributes to the overall electrocatalytic performance.

In this study, we have compared our HHE materials in alkaline environment with other reported OER publications in alkaline media, as shown in Table 3.

The CNT1-RVC and CNT3-RVC materials exhibit strong electrocatalytic activity for the OER relative to many CNT-based systems reported in the literature [44,45,46,47,48]. In 0.2 M KOH, Tafel slopes of approximately 90 mV dec^−1^ were obtained, which are significantly lower than those reported for CoFe_2_O_4_@CNT (229 mV dec^−1^) [43] and NiCo_2_O_4_/CNT (133 mV dec^−1^) [44], and are comparable to the benchmark RuO_2_ catalyst (105 mV dec^−1^) [44]. Two of the nanomaterials, NiFe-functionalized CNTs [46] and heteroatom-doped CNTs [47,48], indicated lower Tafel slopes (70 mV dec^−1^ and 74–76 mV dec^−1^ respectively) and possibly faster kinetics.

While many of the earlier CNT–nanocatalyst materials show promising performance, it must be pointed out that they used dispersed CNTs functionalized with the nanoparticle/atom. The active component (CNT–particle/atom complex) appears to be in loose powdery form and not integrated as a monolithic solid. The commonly used approaches of dispersing powdered nanomaterials seem to be either dispersing them in chemical binders, which are subsequently used to paint or drop-cast on existing electrodes, or loading them into hollow electrodes. In all these methods, the durability and reusability of such nanomaterial complexes for use in practical electrolysis devices may be questionable. Our study is aimed at highlighting a promising alternative architecture of nano electrode design, a robust solid hybrid electrode that shows Tafel slopes comparable to or better than its powdered material counterparts.

The principal advantage of the present architecture lies in its structural integrity and versatility: CNT carpets are directly grown on the three-dimensional RVC scaffold, resulting in a binder-free electrode with continuous conductive pathways, high porosity, and efficient mass transport. Few factors should be considered in designing the HHE materials for future optimization: increasing CNT length can help to some extent but may induce entanglement as mentioned earlier [38]. Similarly, PdNP attachment can be very beneficial in neutral and acidic electrolytes but may partially obstruct OH^−^ adsorption in alkaline media, reducing catalytic efficiency. One important outcome is that this architecture can make it easy to incorporate any metallic nanocatalyst as needed. In earlier studies by this group, palladium loading had been systematically carried out using detailed SEM-EDS studies in combination with other quantitative physical property measurements [33,35]. It was demonstrated that in this processing method, Pd accounts for about 11% the weight of the CNT and the CNT fraction of the overall CNT-RVC sample is about 22%, making the overall mass loading of Pd to be about 2.45 wt%. It could be seen that the PdNP loading is among the lowest levels in comparison with other studies reported in the literature. In our case, the addition of PdNP increases the promise for these electrodes in saline media, which can be very important for practical electrolyzers using seawater. Overall, these results demonstrate the intrinsic advantages of creating robust and reusable electrodes with covalently bonded CNT carpets and suggest that their performance can be further enhanced through heteroatom attachment of transition-metal catalytic sites.

#### 3.2.2. Hydrogen Evolution Reaction (HER)

**Figure 6:**
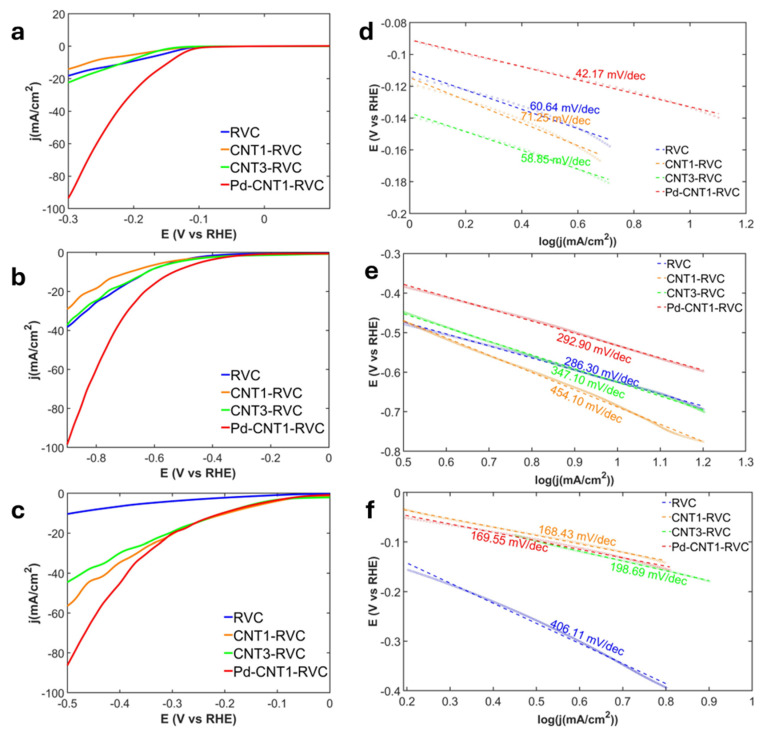
LSV curves of CNT-RVC-based electrodes for HER, taken at a scan rate of 10 mV/s in 0.2 M; (**a**) H_2_SO_4_, (**b**) KCL, and (**c**) KOH. Tafel plots in 0.2 M; (**d**) H_2_SO_4_, (**e**) KCL, (**f**) KOH, extrapolated from the LSV curves.

Figure 6 shows the LSV curves and extrapolated Tafel slopes of the hierarchical hybrid electrodes (HHE) for HER measured in 0.2 M H_2_SO_4_, KOH, and KCl electrolytes. Table 4 summarizes the comparative performance highlighting the onset potential and Tafel slope across different electrolytes.

It must be noted that just as OH^−^ ions are important in facilitating OERs, H^+^ ions play a major role in facilitating HER. In acidic medium, Gibbs free energy is inversely related to the concentration of H^+^ ions, as shown in Equations (9) and (10).

(9) $$2H^{+}+2e^{-}\to H_{2}Q=\frac{pH_{2}}{{\left[H^{+}\right]}^{2}}$$

(10) $$\Delta G_{H_{2}/H_{2O}}=\Delta G^{\circ }+RT\ln\left(\frac{pH_{2}}{{\left[H^{+}\right]}^{2}}\right)$$

In acidic electrolytes such as H_2_SO_4_, the high concentration of H^+^ ions lowers the Gibbs free energy for HER, enabling the reaction to proceed at lower overpotentials. This explains the consistently lower onset potential observed in acidic conditions.

On the other hand, the first step (Equations (11) and (12)) in enabling HER in neutral and alkaline media would be water dissociation to produce the H^+^ ions. This extra step is expected to make the reaction sluggish and explains the higher onset potential in basic and neutral conditions seen in Table 4.

(11) $$2H_{2}O+2e^{-}\to H_{2}+2OH^{-}\;\;Q=\frac{pH_{2}{\left[OH^{-}\right]}^{2}}{{\left[H_{2}O\right]}^{2}}$$

(12) $$\Delta G_{H_{2}/H_{2O}}=\Delta G^{\circ }+RT\ln\left(\frac{pH_{2}{\left[OH^{-}\right]}^{2}}{{\left[H_{2}O\right]}^{2}}\right)$$

The key findings from these results can be summarized as follows:

**Discussion of Onset potential for HER:**

It is seen that the neutral electrolyte needs the highest onset potential. Overall, the onset potential in neutral environments was very comparable in different electrodes, with the PdNP-CNT1-RVC providing minor improvement.

In alkaline medium, addition of CNT to the electrodes provides the most substantial impact. It is reported earlier that in alkaline environment, the availability of hydroxyl ions can directly impact the onset potential [13], as also seen from Equations (11) and (12). As discussed earlier, CNTs have better OH^−^ affinity and can enable a quick accumulation of OH^−^ on the CNT surface, causing the drastic improvement of onset potentials from bare RVC (−0.492 V) to CNT1-RVC (−0.196 V). Longer CNT carpets may not offer additional advantages and may even lead to slight detriment due to possible entanglements. Similarly, adding PdNPs may also be slightly detrimental because they can occupy some active sites on the CNT surfaces, hence impacting OH adsorption.

In acidic environments, Pd-CNT1-RVC electrode showed the lowest onset potential, indicating the easiest reaction initiation. Palladium is known to have a high capacity to adsorb hydrogen, forming palladium hydride (PdH_x_) even at room temperature and atmospheric pressure [49]. This strong hydrogen affinity of Pd nanoparticles most likely accounts for the exceptionally low onset potential of the Pd electrode in acidic media.

**Tafel slopes for HER:**

Figure 6d–f present the Tafel slopes of HER with HHE in acidic, neutral and basic electrolytes, with pH values of 0.7, 7.0 and 13.4. respectively. The Tafel slopes (β) calculated were in terms of mV/dec (mA/cm^2^). Overall, the HHE performance in HER was superior in acidic environments, as expected from the earlier discussion about availability of H^+^ species. The lowest Tafel slope observed was 42.2 mV/dec for Pd-CNT1-RVC electrode in 0.2 M H_2_SO_4_.

The impact of nanocomponents on Tafel slope appears to follow a similar trend as that on onset potential. It is seen that Pd-CNT1-RVC electrode reduced the Tafel slope in acidic and neutral environment significantly. On the other hand, the CNT1-RVC electrode outperformed others in basic condition.

In acidic conditions, hydrogen evolution is dependent on H^+^ adsorption onto the electrode surface to form surface-adsorbed *H species, [50]. Hence, any site that adsorbs hydrogen is expected to have a lower Tafel slope. Since Pd is known to have a high capacity to absorb and store hydrogen spontaneously [51], the PDNP-CNT1-RVC electrode shows the best performance.

**Comparison with HER results in the literature:**

The performance of the Pd-CNT1-RVC electrode seen in this study has been compared with those of other reported publications as shown in Table 5. It must be noted that earlier reported studies were performed in higher electrolyte concentrations, which may spontaneously increase the availability of ions at the reactions site. We have purposely kept electrolytic concentrations at a minimum in our study to reduce caustic environment of the electrolytic cell. Moreover, as mentioned earlier, these are loose powdery CNT–nanocatalyst complexes and not monolithic solids. The powders have been painted or drop-cast on existing electrodes.

The PdCNT1-RVC electrode of this study exhibits a Tafel slope of 42.2 mV per decade in 0.2 M H_2_SO_4_, indicating relatively strong hydrogen evolution activity, with even lower electrocatalyst loading. In comparison, W_2_C supported on CNT showed a higher Tafel slope of 57.4 mV/dec [52], and W_2_C nanoparticles on multiwalled CNTs showed a comparable slope of 45 mV per decade under significantly stronger acidic conditions [53].

Few studies have been reported using Ru, known as the best catalytic metal for HER [10,24,54], attached to CNT-based structures. Ru with MoO_2_ on CNTs, labeled as RMC-500 [55], presents a comparable Tafel slope of 45 mV per decade in 1.0 M KOH. MoC and Mo_2_C hybrids display slopes ranging from approximately 43 to 53 mV per decade in 0.5 M H_2_SO_4_ [56], with variations arising from differences in phase composition and distribution on the CNT support. One study that used Ru nanoparticles supported on multiwalled CNTs and the CNT–catalyst complex drop-casted on an existing electrode [57] showed a lower slope of 27 mV/dec compared to Pd-CNT1-RVC electrode in both strongly acidic (0.5 M H_2_SO_4_) and strongly alkaline (1 M KOH) environments. These studies may point to the future possibility of enhancing the performance of HHE materials by replacing PdNP with other nanocatalysts.

In summary, the variations in Tafel slopes demonstrated by our HHE materials in different electrolytes highlight the strong influence of both the electrode architecture and the surrounding chemical environment on HER activity. In our study, where we are focusing on a monolithic hierarchical hybrid electrode (HHE), the different contributions of CNT and PdNP on the HER in different electrolytes underscores the importance of optimizing the HHE architecture to achieve maximum HER performance for hydrogen production. Future studies to compare how different metal catalysts (such as Ru, Ir and Pt) or emerging nanocatalysts from outside the Pt family can impact the performance of HHEs will be very useful.

**Table 5:** Comparison of CNT-based electrode compositions and other compositions tailored and studied for a better efficiency of HER.

Electrode Composition	Tafel Slope (mV/dec)	Electrolyte	Catalyst Loading	Ref
PdNP-attached carbon nanotube (Pd-CNT1-RVC)	42.2	0.2 M H_2_SO_4_	0.14 mg/cm^2^	This study.
W_2_C@CNT-S8	57.4	0.5 M H_2_SO_4_	NA	[52]
W_2_C nanoparticles on MWNT (W_2_C/MWNT)	45	0.5 M H_2_SO_4_	0.56 mg/cm^2^	[53]
Ru/MoO_2_–CNT (RMC-500)	45	1.0 M KOH	0.416 mg/cm^2^	[55]
MoC–Mo_2_C hybrid	~43–53	0.5 M H_2_SO_4_	0.14 mg/cm^2^	[56]
Ru nanoparticles on MWCNT (Ru@MWCNT)	27	0.5 M H_2_SO_4_, 1 M KOH	0.7 mg/cm^2^ 0.16 mg/cm^2^	[57]

#### 3.2.3. Analysis of OER and HER in Acidic and Alkaline Environment

**Figure 7:**
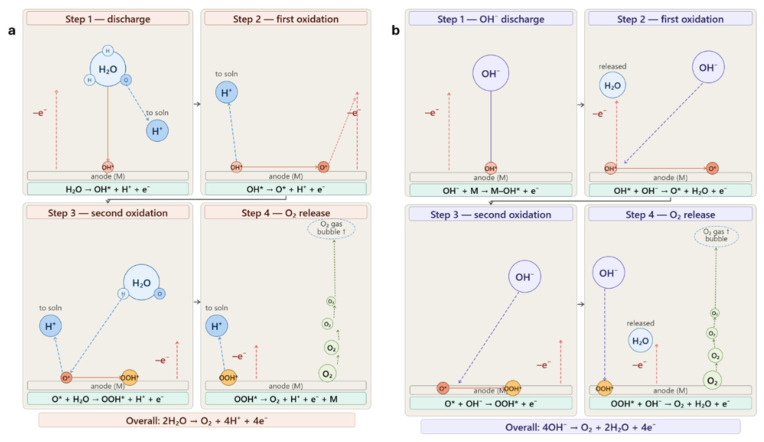
Representation of OER in (**a**) H_2_SO_4_ and (**b**) KOH.

For OER in H_2_SO_4_, the four steps highlight why the initial oxidation is so limited as seen in Figure 7a and Figure S3a,c. In Table S1 it is reported that bare RVC shows a very high slope of 753.8 mV/dec for OH* formation, confirming that carbon cannot effectively stabilize early intermediates like OH* and O* [58]. CNTs make this step even worse, with slopes of 1032 and 946 mV/dec for CNT1-RVC and CNT3-RVC, likely because they occupy surface sites without enabling proper oxidation. However, they do improve the third step, OOH* formation, with slopes around 249 mV/dec and 218 mV/dec, as their π–electron system can assist in O–O bond formation. When PdNPs are introduced, the first step becomes so fast that it is no longer distinguishable, indicating that Pd can effectively stabilize OH* and O* intermediates. The OOH* formation slope drops further to 171.12 mV/dec and O_2_ release to 271.77 mV/dec, showing that PdNPs creates a much more favorable pathway across all steps.

From Figure 7b and Figure S3b,d OER improves in KOH in the early steps because OH^−^ is directly available, lowering the barrier for initial oxidation. This is reflected in the lower slope which is reported in Table S2 for bare RVC at 452.7 mV/dec compared to acid. CNT1-RVC shows a strong improvement, especially in the later steps, with OOH* formation dropping to 84.51 mV/dec and O_2_ release to 169.47 mV/dec, confirming that CNTs are particularly effective at facilitating O–O bond formation and oxygen release. However, CNT3-RVC again shows diminished performance in the first step, with a slope of 609.4 mV/dec, due to aggregation effects. Pd-CNT1-RVC provides the most balanced behavior, with an OH^−^ oxidation slope of 397.0 mV/dec and improved values of 123.6 mV/dec and 253.5 mV/dec for OOH* formation and O_2_ release. This indicates that PdNPs helps stabilize intermediates across all four steps, while CNTs assist in the later stages, resulting in a system where no single step dominates the kinetics.

**Figure 8:**
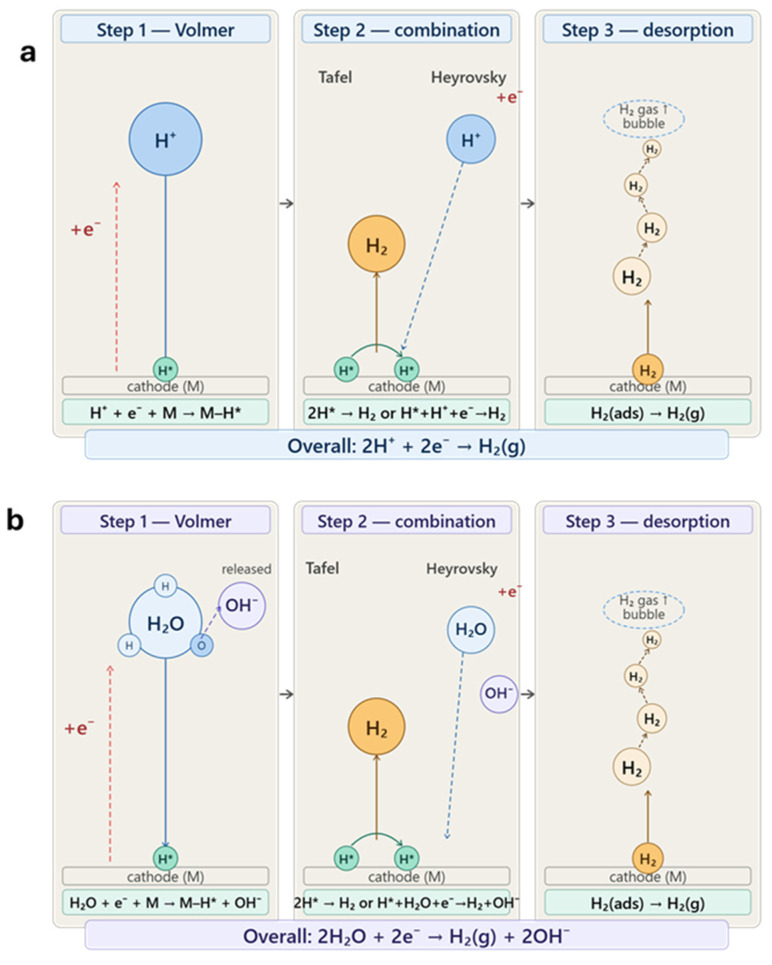
Representation of HER in (**a**) H_2_SO_4_ and (**b**) KOH.

For HER in H_2_SO_4_, the three steps explain both the trends and the numerical results as seen in Figure 8a and Figure S4a,c. As noted in Table S3 in the Volmer step, where protons are reduced to adsorbed hydrogen, bare RVC shows an extremely high slope of 1124 mV/dec, confirming that proton discharge is severely hindered because carbon binds hydrogen too weakly. When CNTs are added, this step improves significantly, as seen from the drop to 76.4 mV/dec for CNT1-RVC, due to defects and edge sites that can stabilize H*. However, increasing the loading to CNT3-RVC worsens the slope to 196.3 mV/dec, which can be explained by CNT aggregation reducing accessible active sites. With Pd-CNT1 RVC, the adsorption slope drops further to 47.5 mV/dec, indicating that this step is no longer limiting. The reaction instead shifts to the combination step, reflected by the ~42 mV/dec slope, where hydrogen atoms recombine to form H_2_. This happens because PdNPs binds hydrogen strongly enough to favor adsorption but makes recombination the slower step. The desorption slope is also lowest at 76.98 mV/dec, confirming that PdNPs enable smooth progression through all three steps.

#### 3.2.4. Stability of Electrodes

**Figure 9:**
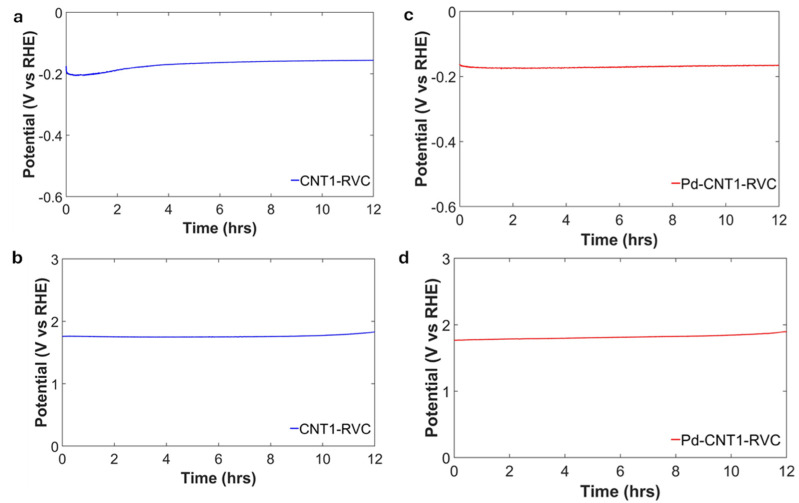
Chronopotentiometric voltage–time profiles of CNT1–RVC and Pd–CNT1–RVC electrodes recorded in (**a**,**c**) 0.2 M H_2_SO_4_ at a current density of −10 mA cm^−2^ and (**b**,**d**) 0.2 M KOH at a current density of 10 mA cm^−2^.

Stability testing of electrodes is essential to evaluate their ability to sustain electrochemical reactions over extended periods without significant degradation. In this study, the results indicate that the Pd-CNT1-RVC electrode exhibited a comparatively lower Tafel slope for the hydrogen evolution reaction in an acidic H_2_SO_4_ environment, suggesting enhanced catalytic activity. In contrast, the CNT1-RVC electrode demonstrated the lowest Tafel slope for the oxygen evolution reaction in an alkaline (KOH) medium. Since these electrodes showed the most promising catalytic performance, stability tests were specifically conducted on them. These tests were performed to assess their durability and operational reliability under their respective working conditions. The chronopotentiometric voltage–time profiles shown in Figure 9 provide insight into the stability of the HHEs under different conditions. The Pd–CNT1–RVC electrode exhibits stable behavior in alkaline media, with less than 2% variation in potential over the initial 12 h, indicating minimal degradation. In acidic conditions, the electrode maintains an almost constant potential throughout the entire duration of the experiment (12 h), further demonstrating its excellent electrochemical stability. The CNT1–RVC electrode shows a gradual increase in potential of up to ~10% during the first 3 h in acidic media, suggesting an initial adjustment or activation process. After this period, the potential stabilizes and remains relatively constant for the remainder of the test (12 h).

## 4. Conclusions

In this study, hierarchical hybrid electrodes have been fabricated by sequential attachment of strongly bonded nanocomponents such as carbon nanotubes (CNT) and palladium nanoparticles (PdNP) on a porous carbon framework, and their electrochemical interactions related to water electrolysis have been evaluated. The results clearly indicate that addition of CNT as well as PdNP can enhance the electrochemical double-layer capacitance (EDLC) of these electrodes as well as increase its exchange current density.

These electrodes have been evaluated for oxygen evolution reaction (OER) and hydrogen evolution reaction (HER) in acidic, alkaline and neutral electrolytic media to understand the contribution of each nanocomponent in different conditions. It is seen that attachment of CNTs can significantly improve OER performance in alkaline environments, due to their strong affinity and high adsorption of hydroxide (OH^−^) species. Moreover, palladium nanoparticles impact the electrochemical activity of HHEs in several significant ways: (i) The presence of Pd lowers the onset potentials (E_on_) for both OER and HER in all pH conditions. Since onset potential reflects the activation energy required for initiating the reaction, a lower E_on_ indicates reduced energy barriers, which can be linked to increased chemical activity of surface sites of the Pd–nanocatalysts. (ii) In neutral and acidic electrolytes, Pd-NP reduces the Tafel slope for OER indicating that it can speed up the charge transfer rates. However, in alkaline environment, Pd may be slowing the reaction by covering some of the bare CNT surface sites, which are known to have strong affinity for the excess OH ions. (iii) Pd nanoparticles seems to increase the tolerance of water electrolysis systems to chlorine ions. This is especially important for future applications in saline or alkaline environments, which are preferred for cost-effective and ecofriendly water electrolysis systems.

These results clarify the contribution of each nanocomponent in different reactions and show that there is significant design space for customizing these materials for application-specific reactor systems in the future. Future tailoring for specific service conditions are possible by changing CNT length, altering the distribution of Pd nanoparticles within the RVC-CNT framework, and exploring the incorporation of alternative nanocatalysts for enhanced electrocatalytic performance. In summary, this HHE architecture shows future promise in creating durable monolithic solid-state electrodes tailored for maximal performance in water electrolyzers for efficient hydrogen production, and for the advancement of renewable and sustainable energy technologies.

## Figures and Tables

**Table 1:** Parameters extrapolated from the CV voltammogram and Tafel plot.

Electrodes	I_0_ (uA/mg)	E_0_ (V)	EDLC (mF/cm^2^)
RVC	0.5395	−0.188	107.14
CNT1-RVC	1.635	−0.355	982.14
CNT3-RVC	266.75	−1.021	1794.66
Pd-CNT1-RVC	75.8	−0.845	1946.43

**Table 2:** Onset potential (E_on_) and Tafel slopes of hierarchical hybrid electrode (HHE) for OER in different electrolytes.

	Onset Potential (V)			Tafel Slope (mV/dec)		
Sample	Acidic	Neutral	Basic	Acidic	Neutral	Basic
RVC	1.872	2.429	2.024	255.6	412.0	273.3
CNT1-RVC	1.824	2.170	1.698	249.2	526.1	84.51
CNT3-RVC	1.899	1.891	1.681	218.0	354.8	88.7
Pd-CNT1-RVC	1.573	1.812	1.617	171.1	157.7	123.6

**Table 3:** Comparison of CNT-based electrodes tailored to improve OER.

Composition	Tafel Slope Values	Electrolyte	Catalyst Loading	Ref
RVC CNT1-RVC CNT3-RVC Pd-CNT1-RVC	273.3 mV/dec, 90.8 mV/dec, 88.7 mV/dec, 123.6 mV/dec.	0.2 M KOH	N/A N/A N/A 0.14 mg/cm^2^	This study.
CNT NiCO_2_O_4_/CNT RuO_2_	256 mV/dec, 133 mV/dec, 105 mV/dec.	0.1 M KOH	0.28 mg/cm^2^ 0.28 mg/cm^2^ 0.2 mg/cm^2^	[44]
CNT CoFe_2_O_4_ @ CNT COFe_2_O_4_	985 mV/dec, 229 mV/dec, 149 mV/dec.	1 M KOH	N/A N/A N/A	[45]
NieFe/TA@CNT, CNT	70 mV/dec, 271 mV/dec.	1 M KOH	0.396 mg/cm^2^ 0.396 mg/cm^2^	[46]
CNT NO-CNT	147 mV/dec, 74 mV/dec.	0.1 M KOH	N/A N/A	[47]
CNT F-doped CNT	75 mV/dec, 76 mV/dec.	1 M KOH	1.9 mg/cm^2^ 1.9 mg/cm^2^	[48]

**Table 4:** Onset potential (E_on_) and Tafel slopes of hierarchical hybrid electrode (HHE) for HER in different electrolytes.

	Onset Potential (V)			Tafel Slope (mV/dec)		
Sample	Acidic	Neutral	Basic	Acidic	Neutral	Basic
RVC	−0.128	−0.626	−0.492	60.6	286.3	406.1
CNT1-RVC	−0135	−0.685	−0.196	71.25	454.1	168.43
CNT3-RVC	−0.215	−0.625	−0.205	58.9	347.1	198.7
Pd-CNT1-RVC	−0.104	−0.533	−0.205	42.2	169.6	292.9

## Data Availability

The original contributions presented in this study are included in the article. Further inquiries can be directed to the corresponding authors.

## Supplementary Materials

The following supporting information can be downloaded at https://www.mdpi.com/article/10.3390/nano16090500/s1.
