# Supplementary material for: Hierarchical Hybrid Electrodes (HHE) for Enhancing the Performance of Water Electrolysis Systems
Source: Nanomaterials (Basel). 2026 Apr 22;16(9):500. doi: 10.3390/nano16090500 (PMC13164860; doi:10.3390/nano16090500)
Supplement: Supplementary file 1 [file nanomaterials-16-00500-s001.zip › nanomaterials-4207849-supplementary.pdf]

Supplementary Documents

Hierarchical Hybrid Electrodes (HHE) for Enhancing the Performance of Water Electrolysis Systems

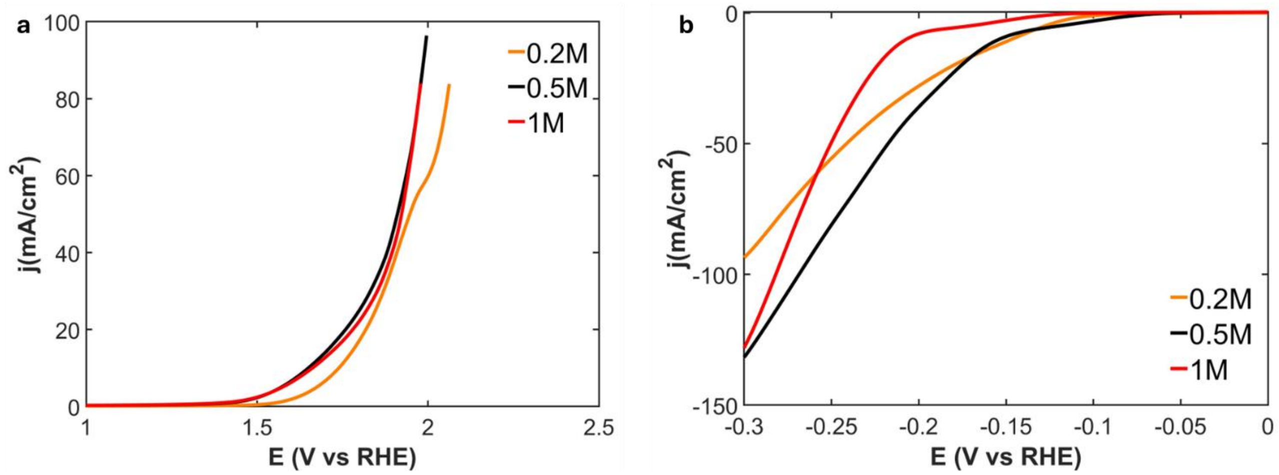

Figure S1: Linear sweep voltammetry (LSV) curves of Pd-CNT1-RVC electrodes for (a) OER and (b) HER, measured at a scan rate of 10 mV s^{-1} in H_2SO_4 electrolytes of varying concentrations (0.2 M, 0.5 M, and 1 M), illustrating the effect of electrolyte concentration on catalytic activity.

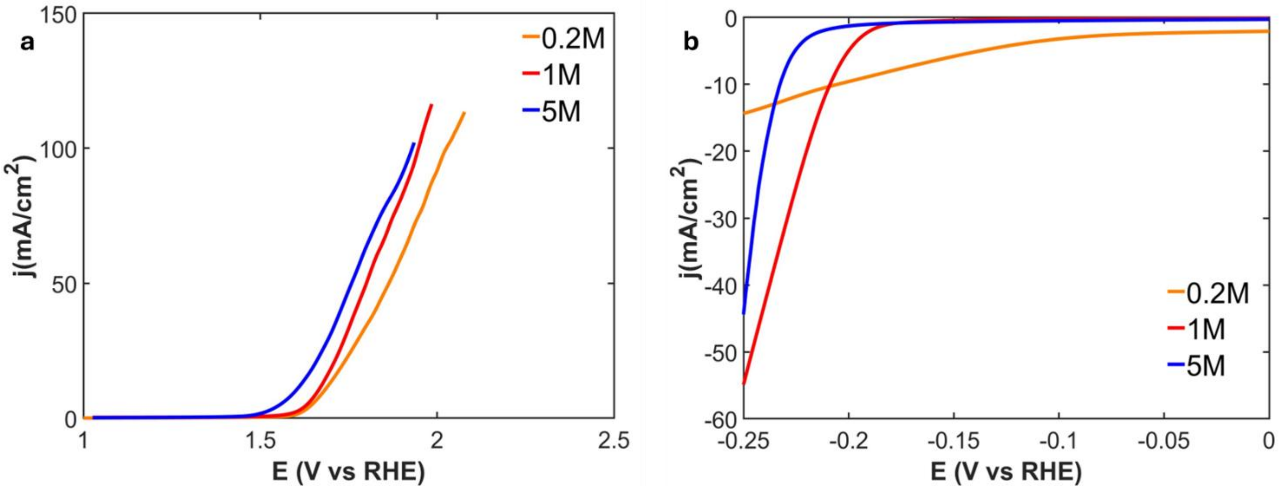

Figure S2: Linear sweep voltammetry (LSV) curves of CNT3-RVC electrodes for (a) OER and (b) HER, measured at a scan rate of 10 mV s^{-1} in KOH electrolytes of varying concentrations (0.2 M, 1 M, and 5 M), illustrating the effect of electrolyte concentration on catalytic activity.

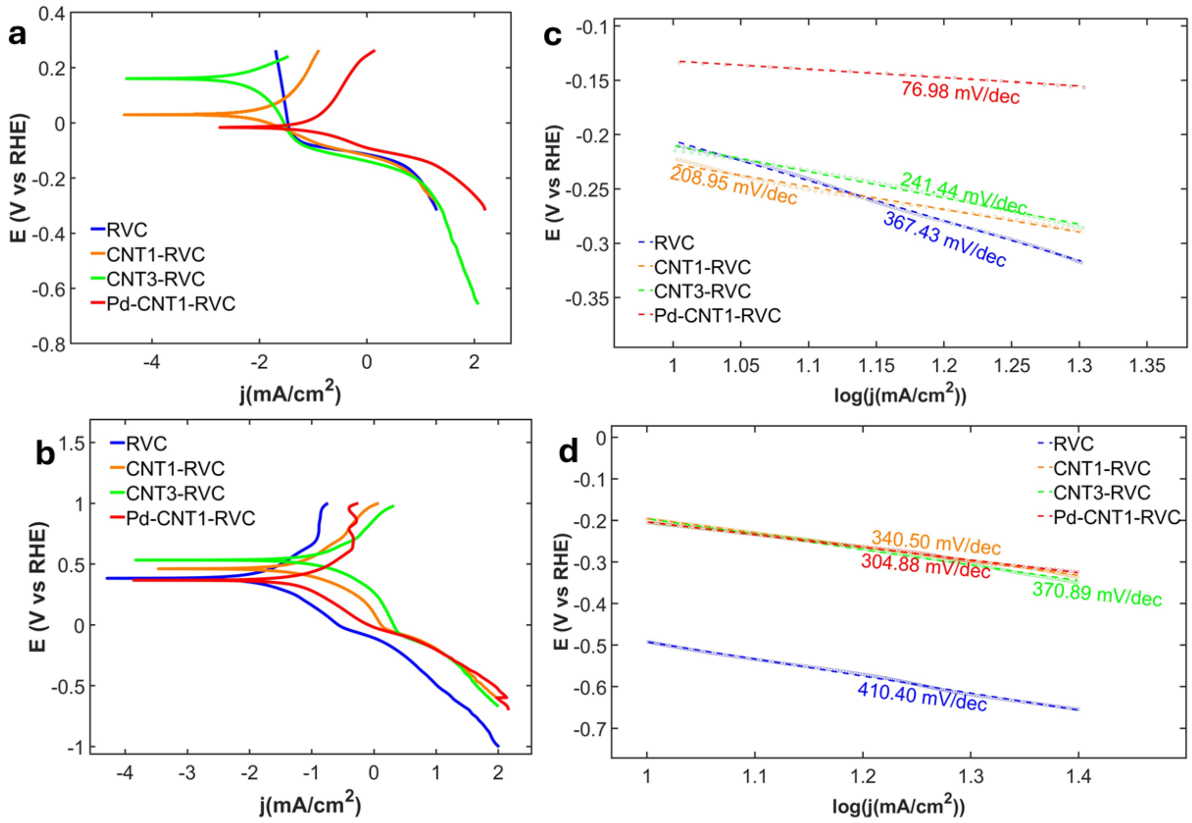

Figure S3: Polarization curves of RVC–CNT-based electrodes for HER measured at a scan rate of 10 mV s^{-1} in (a) 0.2 M H_2SO_4 and (b) 0.2 M KOH, with the corresponding Tafel plots at higher potentials shown in (c) and (d), respectively.

Table S1: Tafel slopes of RVC-CNT based electrodes for HER in 0.2M H_4SO_4

	**Tafel Slopes (mV/dec)**		
**Electrodes**	**Adsorption**	**Combination**	**Desorption**
RVC	1124.0	60.64	367.43
CNT1-RVC	76.4	71.25	208.95
CNT3-RVC	196.3	58.85	241.44

Pd-CNT1-RVC	47.5	42.17	76.98

Table S2: Tafel slopes of RVC-CNT based electrodes for HER in 0.2M KOH

	**Tafel Slopes (mV/dec)**		
**Electrodes**	**H+ Formation**	**Combination**	**Desorption**
RVC	342.5	406.11	410.40
CNT1-RVC	595.5	168.43	340.50
CNT3-RVC	962.0	198.69	370.89
Pd-CNT1-RVC	319.3	169.55	304.88

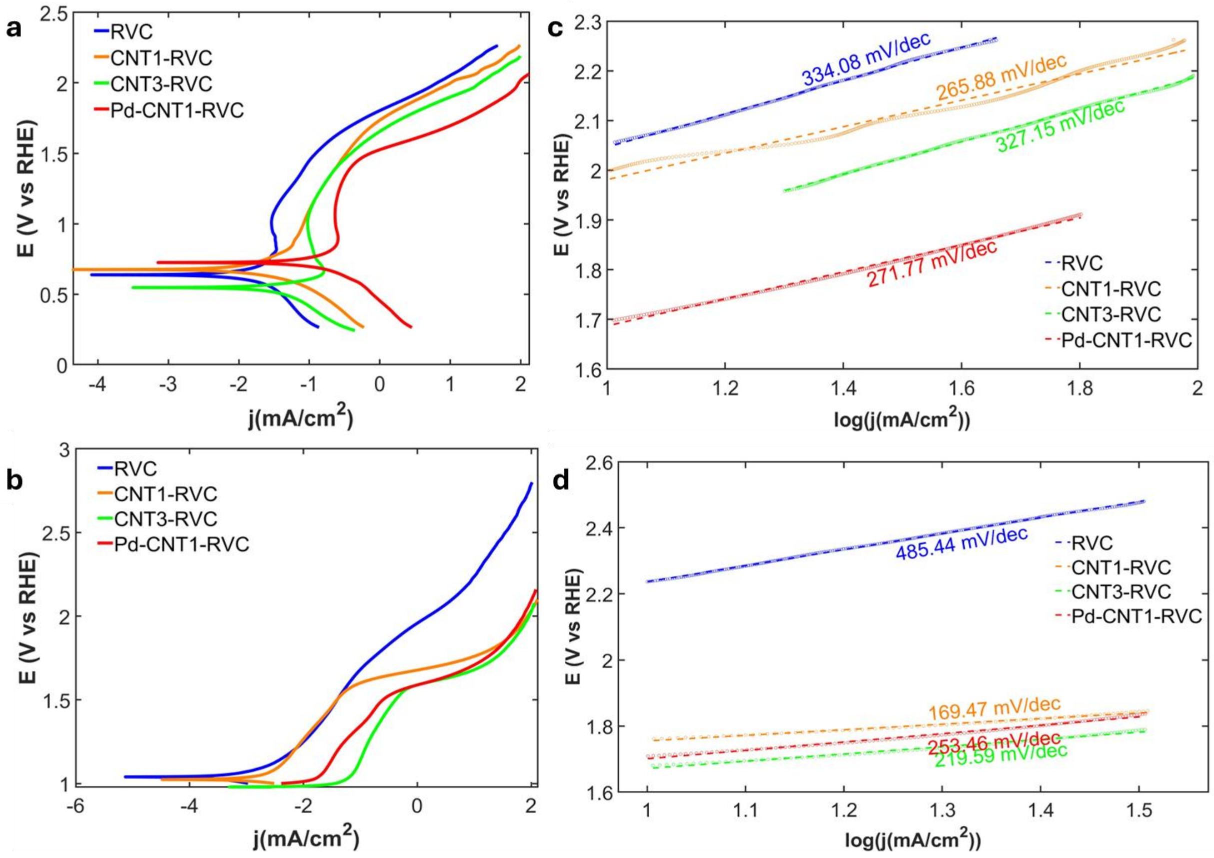

Figure S4: Polarization curves of RVC–CNT-based electrodes for OER measured at a scan rate of 10 mV s^{-1} in (a) 0.2 M H_2SO_4 and (b) 0.2 M KOH, with the corresponding Tafel plots at higher potentials shown in (c) and (d), respectively.

Table S3: Tafel slopes of RVC-CNT based electrodes for OER in 0.2M H_4SO_4

	**Tafel Slopes (mV/dec)**		
**Electrodes**	**OH- Oxidation**	**Formation of *OOH**	**O_2 Release**
RVC	753.8	255.64	334.08
CNT1-RVC	1032.4	249.16	265.88
CNT3-RVC	946.3	218.03	327.15
Pd-CNT1-RVC	--	171.12	271.77

Table S4: Tafel slopes of RVC-CNT based electrodes for OER in 0.2M KOH

	**Tafel Slopes (mV/dec)**		
**Electrodes**	**OH- Oxidation**	**Formation of *OOH**	**O_2 Release**
RVC	452.7	282.09	485.44
CNT1-RVC	393.2	84.51	169.47
CNT3-RVC	609.4	88.70	219.59
Pd-CNT1-RVC	397.0	123.57	253.46
